# Evaluation of Toxicity, Bacteriostatic, Analgesic, Anti-Inflammatory, and Antipyretic Activities of Huangqin-Honghua-Pugongying-Jinyinhua Extract

**DOI:** 10.3390/vetsci8120330

**Published:** 2021-12-15

**Authors:** Dongyang Ye, Jing Sun, Yinqian Li

**Affiliations:** 1 College of Veterinary Medicine, Northwest A&F University, Yangling 712100, China; wintersunwyp@163.com; 2 College of Veterinary Medicine, China Agricultural University, Beijing 100193, China; 3 Lanzhou Institute of Husbandry and Pharmaceutical Sciences, Chinese Academy of Agricultural Sciences, Lanzhou 730050, China; bangbangdajing@163.com

**Keywords:** bacteria, traditional Chinese veterinary medicine, bacteriostatic, analgesic, anti-inflammatory, antipyretic, toxicity

## Abstract

The extensive use of antibiotics has caused the global spread of multidrug-resistant bacteria and genes, seriously reducing antibiotic efficacy and threatening animal and human health. As an alternative, traditional Chinese veterinary medicine (TCVM) was used in this study for its lack of drug resistance and low toxicity. Huangqin-honghua-pugongying-jinyinhua extract (HHPJE), a novel TCVM, consists of the extracts of Huangqin (*Scutellaria baicalensis*), Honghua (*Carthami Flos*), Pugongying (*Taraxacum*) and Jinyinhua (*Lonicerae Japonicae Flos*), and was developed to treat bovine mastitis. In this study, we evaluated the toxicity, bacteriostatic, analgesic, anti-inflammatory, and antipyretic activities of HHPJE. Our results show that HHPJE did not show any acute oral toxicity and can be considered safe for oral administration. Additionally, HHPJE possessed a dose-dependent antibacterial effect on *Staphylococcus aureus*, *Escherichia coli*, *Streptococcus agalactiae* and *Streptococcus dysgalactiae*. HHPJE (60, 30 and 15 g/kg) can reduce the abdominal pain by 44.83 ± 7.69%, 43.15 ± 9.50% and 26.14 ± 4.17%, respectively. The percentages of anti-inflammation inhibition (60, 30 and 15 g/kg) were 35.34 ± 2.17%, 22.29 ± 2.74% and 12.06 ± 3.61%, respectively. The inhibition rates (60, 30 and 15 g/kg) of antipyretic activity were 82.05%, 65.71% and 52.80%, respectively. The evaluation of pharmacodynamics and toxicity indicate that HHPJE possesses significant bacteriostatic, analgesic, anti-inflammatory and antipyretic potential, and also that it is safe for acute oral toxicity, which means it has potential value for treating bovine mastitis in future and alleviating clinical symptoms with no drug resistance or side effects.

## 1. Introduction

Bovine mastitis is mainly caused by bacterial infection, which causes large economic losses for husbandry every year. To date, antibiotics, non-steroidal anti-inflammatory drugs (NSAIDs) or steroidal drugs are the main choices for treating this disease in the clinic. However, the widespread use of antibiotics not only causes drug resistance [1,2]; it also leads to multiple side effects such as allergy, metabolic disorders, and abortion [3,4]. As an alternative, traditional Chinese veterinary medicine (TCVM), with no drug resistance, low toxicity, and rare side effects, has caused much concern [5,6]. TCVM possesses lots of pharmacological effects such as antioxidation, bacteriostasis, and anti-inflammation. In addition, some TCVMs can improve immunity and kill tumor cells [7,8].

Huangqin-Honghua-Pugongying-Jinyinhua extract (HHPJE), a novel TCVM formula, consists of extracts of Huangqin (*Scutellaria baicalensis*), Honghua (*Carthami Flos*), Pugongying (*Taraxacum*) and Jinyinhua (*Lonicerae Japonicae Flos*), and was developed to treat bovine mastitis. Orthogonal test and single factor test were used to optimize the extraction methods and the best proportion of each ingredients of HHPJE in our previous study. Four primary extracted components, including baicalin, hydroxy safflower yellow A, chlorogenic acid and caffeic acid, were extracted and analyzed by high performance liquid chromatography (HPLC). The combination of four herbal extracts possesses good synergistic bacteriostasis. Baicalin, a flavonoid, has been proven to be the most abundant component and primary bioactive substance in *Scutellaria baicalensis*, possessing great potential for anti-inflammation, anti-bacteria and anti-virus [9,10]. The chlorogenic acid extracted from Taraxacum has SOD-like activity which plays a vital role in balancing oxidation and antioxidation [11], possessing bacteriostasis, anti-inflammatory, and antiviral effects. *Carthami Flos* possesses the effects of promoting blood circulation, removing blood stasis and relieving pain. Modern pharmacological research shows that it has anti-oxidation and anti-apoptosis effects. Hydroxy safflower yellow A, one of the main components of *Carthami Flos*, activates blood circulation, clearing blood stasis and relieving pain [12,13]. Related research has also proven that it can promote the repair of necrotic tissue and improve blood circulation. Caffeic acid possesses biological activities such as antioxidant, anti-inflammation and anti-bacteria.

In this study, a new TCVM extract formula with multiple pharmacodynamics was proposed for systematically treating bovine mastitis. HHPJE was developed to combine the active substances and achieve advantageous therapeutic effects. Acute toxicity and 28-day sub-chronic toxicity tests were performed firstly, and mice and SD rats survived within the good mental state and normal behavior. No abnormal pathological and histopathological changes were observed. After ensuring the safety of the extract, significant bacteriostatic, analgesic, anti-inflammatory, and antipyretic effects were observed, indicating that HHPJE has potential value in treating bovine mastitis in future and alleviating the clinical symptoms with no drug resistance and side effects.

## 2. Materials and Methods

### 2.1. Drugs and Reagents

Huangqin (*Scutellaria baicalensis*), Honghua (*Carthami Flos*), Pugongying (*Taraxacum*) and Jinyinhua (*Lonicerae Japonicae Flos*) were all provided by Gansu Mengji Pharmaceutical Technology Co., Ltd., and all the TCVMs were identified according to the identification standard of the Pharmacopeia of the People’s Republic of China (Committee, 2015). This includes MH agar, MH broth, BHI broth (Beijing landbridge biotechnology Co., Ltd., Beijing, China), antondine injection (Beiliu Tongzhou Pharmaceutical Co., Ltd., Yulin, China), acetic acid (Chengdu Kelong Chemical Reagent Co., Ltd., Chengdu, China), aspirin (AstraZeneca pharmaceutical Co., Ltd., Taizhou, China), xylene (Tianjin bodi chemical Co. Ltd., Tianjin, China), dried yeast (Angel yeast co. Ltd., Yichang, China), and paracetamol (Southwest pharmaceutical Co. Ltd., Chongqing, China).

### 2.2. Animals and Housing

Female and male Kunming mice (4 weeks old) were purchased from the Experimental Animal Center of Xi’an Jiaotong University (laboratory animal reproduction license #SYXK Shan 2012-005). All animals were arbitrarily housed at the Veterinary Pharmacology and Toxicology Lab of Northwest A & F University for one week to acclimate to the laboratory environment before starting the experiment. They were housed in a standard environmental condition with well-balanced rodent diets and fresh tap water ad libitum. The laboratory temperature was maintained at 22 °C (±3 °C), and the relative humidity was 50–60%. During the whole test period, the light/dark cycle time was 12 h, and the illumination was 160–290 lux. All animal procedures and study design were conducted in accordance with the Guide for the Care and Use of Laboratory Animals (Ministry of Science and Technology of China), and were approved by the animal ethics committee of Northwest A & F University (Ethical number: NWLA-2020-0118).

### 2.3. Acute Toxicity and 28-Day Sub-Chronic Toxicity

Acute oral toxicity was designed according to the Organization of Economic Cooperation and Development (OECD) guidelines for testing chemicals, guideline 425: Acute Oral Toxicity–Up-And-Down Procedure (OECD, 2006). Kunming mice (18.0–22.0 g) were selected and randomly divided into seven groups, with six mice in each group of both sexes. Groups I to VI were administered with HHPJE at 80, 60, 40, 30, 20, and 10 g/kg, and group VII was gavaged with normal saline at 30 mL/kg. Clinical observations were monitored and recorded for 48 h, including general observations, water intake, food intake, toxicological signs, and mortality. The sub-chronic were designed according to OECD Guideline 408. Eighty SD rats were randomly divided into four groups (60, 30, 15 g/kg HHPJE and normal saline), 20 rats per group with 10 females and 10 males, respectively. Mental state, breathing and behavior were observed twice daily during the study. At the end of the study, histological examinations were performed. 

### 2.4. Bacteriostatic Activity

#### 2.4.1. Oxford Cup Method

*Escherichia coli*, *Staphylococcus aureus*, *Streptococcus agalactiae* and *Streptococcus dysgalactiae*, which were all isolated in the clinic, were used. The bacterial cultures were uniformly coated on the surface of medium. 2.0 g/mL, 1.0 g/mL and 0.5 g/mL of HHPJE were added to the Oxford Cup and cultured for 18 h at 37 °C. The diameters of bacteriostatic zones were measured by vernier calliper. Three biological replicates were prepared. 

#### 2.4.2. MIC and MBC

The MIC and MBC were determined using the broth microdilution method following the recommendations given in the Clinical and Laboratory Standards Institute (CLSI) documents VET01-S3 and M100-S25 (CLSI., 2015).

### 2.5. Analgesic Activity 

#### 2.5.1. Acetic Acid-Induced Writhing Test

The analgesic activity of HHPJE was evaluated using the acetic acid-induced writhing test in line with Faiz Hossain et al.’s method [14]. Kunming mice (18.0–22.0 g) (*n* = 10) were randomized into five groups of 10 mice each. All animals were fasted overnight before the test. Group I was gavaged with normal saline (30 mL/kg) as control, Group II was injected with antondine injection (5 mL/kg) while the remaining three groups were gavaged with 60, 30 and 15 g/kg HHPJE, respectively. After one h, the mice were treated with acetic acid (0.6%, *v/v* in saline, 10 mL/kg b w., i.p.) to induce pain sensation. The reaction of the mice within 20 min was carefully observed, and the writhing reactions were characterized by intermittent convulsions, abdominal constrictions, and the extension of hind limbs or crossing. The number of writhing reactions for each mouse was recorded within 20 min after acetic acid injection. The percentage of analgesic inhibition was calculated as follows:

$$\%\mathrm{Inhibition}=\frac{\mathrm{number}\mathrm{of}\mathrm{writhes}(\mathrm{control})-\mathrm{number}\mathrm{of}\mathrm{writhes}(\mathrm{test})}{\mathrm{number}\mathrm{of}\mathrm{writhes}(\mathrm{control})}\times 100$$

#### 2.5.2. Hot Plate Test

The hot plate test was performed according to a previously described method by Zhang et al. [15]. Female Kunming mice (18.0–22.0) were domesticated under laboratory conditions one hour before the experiment. The hot plate device consisted of a water bath and a glass cylinder (diameter 15 and 20 cm high), and the temperature of the glass cylinder was maintained at 55 ± 0.1 °C. After mice were placed on the heated plate, the time of the first lick of the foot was the latency time of mice. Animals were subjected to a pre-testing, and the mice with a latency time longer than 30 s and less than 5 s during pre-testing were excluded. Then, all the selected animals were randomized into five groups (with 10 mice each). Group I was gavaged with normal saline (30 mL/kg) as control. Group II was injected with antondine injection (5 mL/kg) while the remaining groups III, IV and V were gavaged with 60, 30 and 15 g/kg HHPJE, respectively. After 30 min, 60 min, 90 min and 120 min, the latency time was measured in seconds. The cut-off time was 60 s to protect the tissues of mice [16,17]. The percentage of analgesic inhibition was calculated as follows:

$$\%\mathrm{Inhibition}=\frac{\mathrm{Post-treatment}\mathrm{latency}-\mathrm{Pre-treatment}\mathrm{latency}}{\mathrm{Cut-off time}-\mathrm{Pre-treatment}\mathrm{latency}}\times 100$$

### 2.6. Anti-Inflammatory Activity 

The anti-inflammatory activity of HHPJE was determined by the method of auricular oedema induced by xylene, as described by Chen et al. [18]. Mice (18.0–22.0 g) were randomly allotted to 5 groups of 10 animals each. Ninety minutes after oral treatment of each group with normal saline (30 mL/kg), aspirin (0.2 g/kg) and HHPJE (60, 30 and 15 g/kg), oedema was induced by instilling 30 μL of xylene to the anterior and posterior surfaces of the right ear whereas the left ear considered as control. After 30 min of xylene application, a metal punch was used to obtain ear tissue. The weights of the left and right ear in each group were measured, and the inhibition rates were calculated using the following formula:

$$\%\mathrm{Inhibition}=\frac{\mathrm{increase}\mathrm{in}\mathrm{ear}\mathrm{weight}(\mathrm{control})-\mathrm{increase}\mathrm{in}\mathrm{ear}\mathrm{weight}(\mathrm{test})}{\mathrm{increase}\mathrm{in}\mathrm{ear}\mathrm{weight}(\mathrm{control})}\times 100$$

### 2.7. Antipyretic Test

Kunming mice (18.0–22.0) were used to study the antipyretic activity of HHPJE by the yeast-induced pyrexia method [19]. The selected animals were healthy and adapted to laboratory conditions at the start of the experiment. All mice were fasted for one night, but free water was allowed. After 24 h, the rectal temperature of mice in each group was measured by the digital thermometer, and then 20% yeast water suspension was injected to cause pyrexia (10 mL/kg). Then, the rectal temperatures were measured at 18 h again after yeast injection. Mice with a temperature increase above 0.5 °C were confirmed to be feverish, while those with a temperature increase below 0.5 °C were discarded. Male and female mice were randomly divided into five groups of 10 mice each. Group I was gavaged with normal saline (30 mL/kg) as control, Group II was gavaged with paracetamol (150 mg/kg), while the remaining groups III, IV and V were gavaged with 60, 30 and 15 g/kg HHPJE, respectively. The temperatures of the rectum were recorded at 30, 60, 90, 120, 150, 180 min after drugs administration. The inhibition rates were calculated using the following formula:

$$\%\mathrm{Inhibition}=\frac{B-\mathrm{Cn}}{B-A}\times 100$$

A represents normal body temperature; B represents temperature after pyrexia induction; Cn represents temperature after 30, 60, 90, 120, 150, 180 min. 

### 2.8. Statistical Analysis

Data were expressed as mean ± SD. The values were analyzed by one-way ANOVA followed by multiple comparisons, and effects were considered significantly different at the *p* < 0.05 level while extremely significantly different at the *p* < 0.01 level. Statistical analysis and figures were carried out using Graphpad prism 8.0 software (GraphPad Software Inc., San Diego, CA, USA). 

## 3. Results

### 3.1. Toxicity 

**Figure 1:**
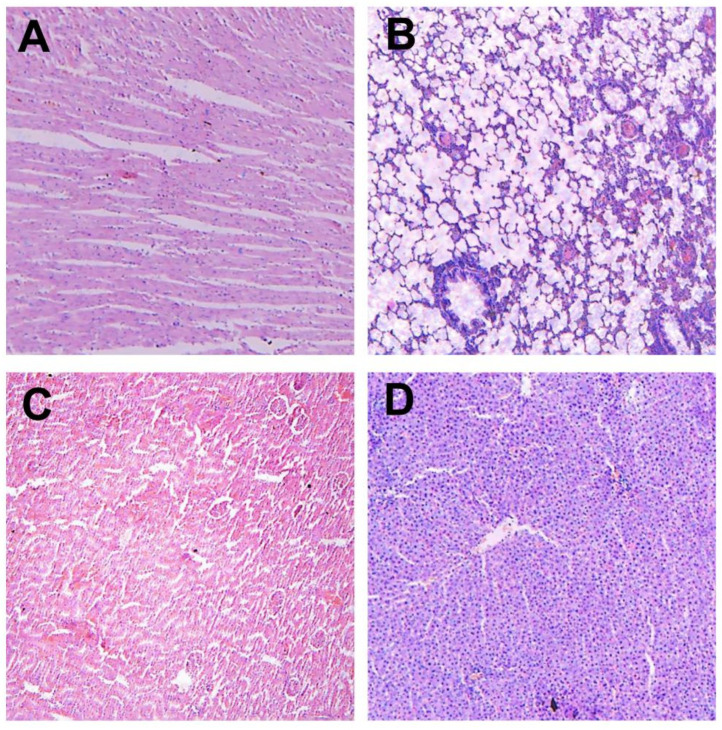
Histological examination after 28-day sub-chronic toxicity test. (**A**) myocardium (100×); (**B**) lung (100×); (**C**) kidney (100×); (**D**) liver (100×).

HHPJE did not show any acute and sub-chronic toxicity during administration. Mice and SD rats in all groups survived within the good mental state and normal behavior. No abnormal pathological changes were observed in the heart, liver, spleen, lung, kidney, stomach, intestines and other major organs of mice. No histopathological changes were observed after the 28-day sub-chronic toxicity test (Figure 1). Hence, HHPJE can be considered safe for oral administration. 

### 3.2. Bacteriostatic Activity

**Figure 2:**
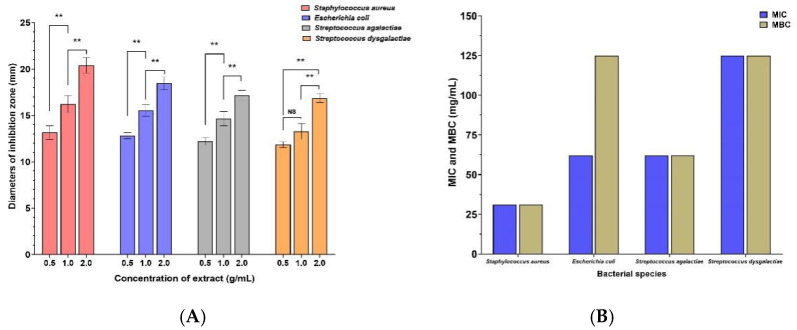
Bactericidal and antibacterial activities of HHPJE. (**A**) MIC of HHPJE on *Staphylococcus aureus*, *Escherichia coli*, *Streptococcus agalactiae* and *Streptococcus dysgalactiae*. (**B**) MBC of HHPJE on 4 strains. Values were expressed as the mean ± SD, and all data were analyzed using one-way ANOVA followed by multiple comparisons. ** represents extremely significantly different between two groups (*p* < 0.01), while NS means no significant differences (*p* > 0.05).

HHPJE showed antibacterial and bactericidal effects on *Staphylococcus aureus*, *Escherichia coli*, *Streptococcus agalactiae* and *Streptococcus dysgalactiae*. For the Oxford Cup method, HHPJE has dose-dependent antibacterial effects on the four strains, and the best antibacterial effect was showed on *Staphylococcus aureus* (Figure 2A). The antibacterial and bactericidal effect of *Staphylococcus aureus* was the best; the Minimum Inhibitory Concentration (MIC) and Minimum Bactericidal Concentration (MBC) were both 31.25 mg/mL (Figure 2B). 

### 3.3. Analgesic Activity 

#### 3.3.1. Acetic Acid Induced Writhing Test 

**Figure 3:**
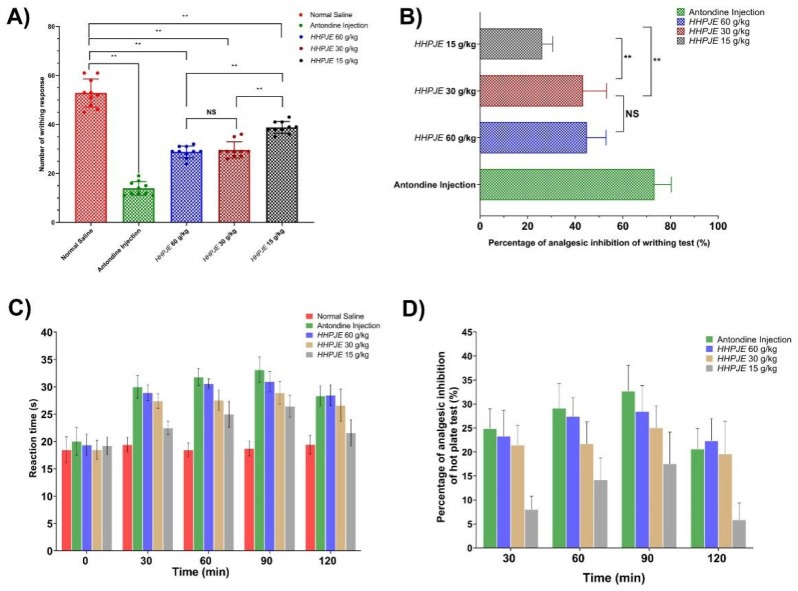
Analgesic activity of HHPJE. (**A**) Change in writhing response induced by acetic acid; (**B**) Percentage of analgesic inhibition of writhing test; (**C**) Changes in reaction time within 0 to 120 min in hot plate test; (**D**) Percentage of analgesic inhibition of hot plate test. Values were expressed as the mean ± SD, and all data were analyzed using one-way ANOVA followed by multiple comparisons. ** represents extremely significantly different between two groups (*p* < 0.01), while NS means no significant differences (*p >* 0.05).

The accumulation of abdominal contraction is related to the level of pain caused by acetic acid. This study revealed that HHPJE reduced the writhing responses induced by injection of 0.6% acetic acid solution, and the relief of pain showed a dose-dependent fashion at all test doses (60, 30 and 15 g/kg i.g.) as shown in Figure 3A. Intragastric administration of HHPJE (60, 30 and 15 g/kg) and intraperitoneal administration of antondine injection in mice significantly reduced the number of writhing response (*p* < 0.01). HHPJE (60, 30 and 15 g/kg) and antondine injection have extremely significantly different results from the normal saline group (*p* < 0.01). HHPJE at 60, 30 and 15 g/kg reduced the abdominal pain by 44.83 ± 7.69%, 43.15 ± 9.50%, 26.14 ± 4.17%, respectively. HHPJE (60 g/kg) and HHPJE (30 g/kg) have extremely significantly different results from HHPJE (15 g/kg) (*p* < 0.01), while the writhing responses of HHPJE (60 g/kg) and HHPJE (30 g/kg) were found to have a non-significant difference (*p* > 0.05). Antondine injection (5 mL/kg) used as a positive control also showed extremely significant inhibition (73.22 ± 6.73%, *p* < 0.01) in the number of writhes as compared to the normal saline group (Figure 3B). 

#### 3.3.2. Hot Plate Test

The results of the hot plate test showed that the latency time and the percentage of analgesic inhibition were dose-dependent at 15, 30 and 60 g/kg. The analgesic activity increased from 0 to 90 min for all the test groups as shown in Figure 3C, while it decreased a little at 120 min. For HHPJE groups, the highest increase in latency time was observed 31.0 ± 1.8 s at 60 g/kg dose at 90 min, whereas the percentage of inhibition also reached 28.4% at this time point (Figure 3D).

### 3.4. Anti-Inflammatory Activity 

**Figure 4:**
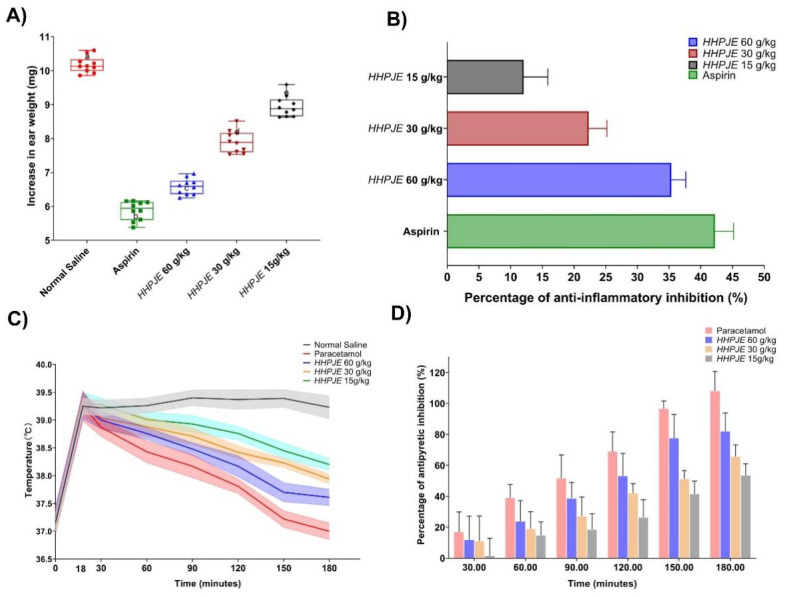
Anti-inflammatory and antipyretic activities of HHPJE. (**A**) The increase in ear weight induced by xylene; (**B**) Percentage of anti-inflammatory inhibition; (**C**) Changes in body temperature during 0 to 180 min; (**D**) Percentage of antipyretic inhibition.

The anti-inflammatory activity of xylene-induced ear oedema showed a dose-dependent inhibition (Figure 4A). According to these results, the anti-inflammatory activities of Aspirin, HHPJE (60 g/kg), HHPJE (30 g/kg), HHPJE (15 g/kg) have an extremely significant difference between normal saline (*p* < 0.01); the increases in ear weight are 6.58 ± 0.23 mg, 7.91 ± 0.31 mg and 8.95 ± 0.29 mg, respectively (Figure 4A). HHPJE (60, 30 and 15 g/kg i.g.) administration inhibited the weight gain by 35.34 ± 2.17%, 22.29 ± 2.74% and 12.06 ± 3.61%, respectively. The positive control aspirin also showed significant inhibition (42.23 ± 2.79%, *p* < 0.01) compared to the normal saline group (Figure 4B). 

### 3.5. Antipyretic Activity 

Antipyretic activity of HHPJE on mice using yeast-induced pyrexia model is shown in Figure 4C,D. After yeast was injected, hyperthermia was produced and maintained at 30, 60, 90, 120, 180 min in the control group. Single oral doses (60, 30 and 15 g/kg) of HHPJE significantly inhibited (*p* < 0.01) yeast-induced pyrexia in mice (Figure 4C). At 30 min post-administration, 60, 30, and 15 g/kg doses of HHPJE, respectively caused average inhibition rates of 11.95%, 11.13% and 0.86% in yeast-induced pyrexia, while this later improved to 82.05%, 65.71% and 52.80% at 180 min when compared with the normal saline group, and the inhibition was dose-dependent (Figure 4D). Paracetamol (150 mg/kg) used as positive control demonstrated an average inhibition rate 17.07% at 30 min to 108.09% at 180 min (Figure 4D).

To sum up, as a novel TCVM formula, HHPJE possesses significant bacteriostatic, analgesic, anti-inflammatory, and antipyretic effects and is safe for oral toxicity. From a pharmacodynamic perspective, HHPJE has potential value in treating bovine mastitis in future and alleviating the clinical symptoms.

## 4. Discussion

The extensive use of antibiotics, NSAIDs, and steroidal drugs in clinics has caused drug resistance and multiple side effects such as allergy, metabolic disorders, infection, and abortion to humans and animals. TCVM, derived from plants or herbs, have no drug resistance, low toxicity and rare side effects [6,20,21]. One TCVM usually has merely one or two pharmacological effects. However, when several TCVMs are jointly used, the synergistic effects may occur and exhibit comprehensive pharmacological effects. Researching novel TCVM formula is a vital direction of new drug development. Our lab screened out TCVM with bacteriostatic, analgesic, anti-inflammatory and antipyretic effects from hundreds of TCVMs and finally determined *Scutellaria baicalensis*, *Carthami Flos*, *Taraxacum*, and Lonicera japonica Thunb as the ingredients of HHPJE based on the syndrome differentiation theory and synergistic antibacterial effects. As a new TCVM formula, the pharmacodynamics and toxicity need to be evaluated before its clinic use. 

The safety of toxicity is a prerequisite for studying pharmacodynamics [22]. HHPJE did not show any acute and sub-chronic toxicity and can be considered safe for oral administration. The pharmacological activities of HHPJE are baicalin, hydroxy safflower yellow A, chlorogenic acid and caffeic acid, which have been reported no toxicity [23,24]. Among the multitudinous aetiologies that cause bovine mastitis, the mammary gland infection by various bacteria is the most important [25]. Hence, antimicrobial activity is an important part of evaluating the pharmacodynamics of HHPJE. HHPJE at all doses (0.5, 1.0 and 2.0 g/mL) possesses antibacterial and bacteriostatic activity on *Escherichia coli*, *Staphylococcus aureus*, *Streptococcus agalactiae* and *Streptococcus dysgalactiae*. Caffeic acid and chlorogenic acid possess antimicrobial activities [26]. Baicalin can protect mice against Salmonella typhimurium infection via the modulation of bacterial virulence and host response [27]. Peng et al. [28] reported that baicalin could inhibit avian pathogenic *Escherichia coli* by inhibiting quorum sensing and inflammatory response. In addition, baicalin possesses antiviral effects by inhibiting viral mRNA [29]. 

Ache can be divided into physical pain and chemical pain. The hot plate method belongs to physical pain, inducing pain through heat stimulation, while the writhing test produces pain through chemical stimulation [30,31]. Our results showed that HHPJE had a significant analgesic effect (*p* < 0.01) on the number of writhe responses induced by acetic acid in a dose-dependent manner, reducing the abdominal pain by 44.83 ± 7.69%, 43.15 ± 9.50%, 26.14 ± 4.17% at 60, 30 and 15 g/kg, respectively. This is mainly because HHPJE can inhibit the releasing of mediators such as bradykinins, substance P, prostaglandins, and pro-inflammatory cytokines such as IL-1, IL-6, IL-8 and TNF-a, which activate the chemosensitive nociceptors. For instance, baicalin, which is extracted from *Scutellaria baicalensis*, can inhibit inflammatory mediators such as Nitric oxide (NO), PGE-2, pro-inflammatory cytokines, and the infiltration of neutrophils in the inflammatory site [32]. HHPJE significantly improved the nociceptive threshold measured by the increased latency time. The analgesic activity increased from 0 to 90 min in all the test groups and decreased a little at 120 min. The highest increase in latency time was observed at 31.0 ± 1.8 s at 60 g/kg at 90 min, and the percentage of inhibition was 28.4%. The reduction in writhing reactions and increasing latency time may be closely related to the reduction in neurotransmitters and inhibition on specific pathways. NO, which is mainly synthesized by inducible nitric oxide synthase (iNOS) and regulated by MAPK/p38 signaling pathway [33,34], is an important second messenger and neurotransmitter, and involved in pain modulation, promoting the transmission of pain and the release of pain mediators [35]. Hydroxysafflor yellow A, one of the main components of *Carthami Flos*, has been shown to inhibit the MAPK/p38/iNOS pathway to reduce the release of NO to relieve pain [36]. In addition, chlorogenic acid, the most abundant polyphenol compound extracted from *Taraxacum* and Lonicera japonica Thunb, was proven to exert analgesic action by modulating acid-sensing ion channels in the primary afferent neurons [37]. Our results indicated that HHPJE might exert analgesic effects by inhibiting the release of NO, PGE-2 and pro-inflammatory cytokines and regulating the signaling pathways associated with the pain response. 

Xylene-induced swelling can induce the release of TNF-α, IL-6 or other cytokines and inflammatory mediators to stimulate the development of inflammation [38]. To date, many TCVMs are proven to have anti-inflammatory activities. For instance, flavonoids isolated from *Scutellaria baicalensis* have significant anti-inflammatory effects in respiratory tract inflammation, inflammatory bowel disease, rheumatoid arthritis, encephalomyelitis, mastitis and other disease models, and are proven to be a promising anti-inflammatory bioactive substance with almost no toxicity to epithelial, peripheral and myeloid cells. Baicalin can inhibit the expression of the proinflammatory cytokines TNF-α, IL-β, and IL-6 by reducing NF-κB pathway, p38 phosphorylation and mRNA expression in *Staphylococcus aureus* mastitis [39]. Furthermore, baicalin can inhibit Salmonella typhimurium-induced inflammation and it mediates autophagy through the TLR4/MAPK/NF-kappa B signaling pathway [40]. Caffeic acid, a vital component in HHPJE, can inhibit the activation of NF-κB and STAT-3, thereby inhibiting the transcription of inducible NO synthase (iNOS), interleukin-6 (IL-6) and TNF-α activation, and ultimately attenuating the production of nitric oxide (NO), IL-6 and TNF-α [41]. Additionally, luteolin, one of the flavonoids extracted from *Lonicerae Japonicae Flos*, significantly inhibited the induction of inflammatory cytokines such as TNF-α, IL-6, IL-8, granulocyte-macrophage colony-stimulating factor and COX-2 through a decrease in the intracellular Ca^2+^ levels, and also showed inhibition of the activation of ERK, JNK and NF-κB [42]. 

Pyrexia is caused by infection, tissue damage, inflammation, malignancy or other diseases. Injection of yeast suspension can cause fever in animal bodies, and the pyrogenic substances are capsular polysaccharides and proteins contained in yeast [43]. Yeast can activate endogenously thermophilic cells to produce and release pyrogenic cytokines, causing an increase in body temperature [44]. In this experiment, mice were injected with yeast suspension for fever modelling. Eighteen hours after the yeast was injected, the control group maintained hyperthermia within 30, 60, 90, 120, 150 and 180 min. HHPJE significantly inhibited(*p* < 0.01) yeast-induced pyrexia in mice at all test doses (Figure 4C). At 30 min post-administration, 60, 30 and 15 g/kg of HHPJE, respectively caused average inhibition rates of 11.95%, 11.13% and 0.86%, while these later improved to 82.05%, 65.71% and 52.80% at 180 min (Figure 4D). PGE-2 is one of the most important central fever mediums. PGE-2 in the central nervous system mainly comes from cerebrovascular endothelial cells and surrounding macrophages and acts in the paracrine form on PGE-2 receptors in hypothalamus temperature-sensitive neurons. Baicalin extracted from *Scutellaria baicalensis* can reduce PGE-2 in the hypothalamus and peripheral blood, which possesses an antipyretic effect [45]. Additionally, the chlorogenic acid possesses high content and antipyretic property in *Lonicera japonica* Thunb [46,47].

## 5. Conclusions

In conclusion, HHPJE is safe for oral toxicity. As a novel TCVM formula, HHPJE combined the extracts of Huangqin (*Scutellaria baicalensis*), Honghua (*Carthami Flos*), Pugongying (*Taraxacum*) and Jinyinhua (*Lonicerae Japonicae Flos*) through orthogonal experiments and was found to possess significant bacteriostatic, analgesic, anti-inflammatory, and antipyretic effects, which have potential in systematically treating bovine mastitis in future and alleviating the clinical symptoms with no drug resistance and side effects.

## Data Availability

The raw data supporting the conclusions of this article will be made available by the authors.
